# Antifungal Activity of Human Cathelicidin LL-37, a Membrane Disrupting Peptide, by Triggering Oxidative Stress and Cell Cycle Arrest in *Candida auris*

**DOI:** 10.3390/jof8020204

**Published:** 2022-02-20

**Authors:** Irfan A. Rather, Jamal S. M. Sabir, Amer H. Asseri, Sajad Ali

**Affiliations:** 1 Department of Biological Sciences, Faculty of Science, King Abdulaziz University (KAU), Jeddah 21589, Saudi Arabia; jsabir@kau.edu.sa; 2 Centre of Excellence in Bionanoscience Research, King Abdulaziz University (KAU), Jeddah 21589, Saudi Arabia; 3 Biochemistry Department, Faculty of Science, King Abdulaziz University (KAU), Jeddah 21589, Saudi Arabia; ahasseri@kau.edu.sa; 4 Department of Biotechnology, Yeungnam University, Gyeongsan 385541, Korea

**Keywords:** *Candida auris*, infection, cathelicidin LL-37, cell cycle arrest, combination therapeutics

## Abstract

*Candida auris,* an evolving multidrug-resistant pathogenic yeast, is known for causing severe invasive infections associated with high mortality rates in hospitalized individuals. Distinct from other *Candida* species, *C. auris* can persist for longer periods on different surfaces and is resistant to all of the major classes of antifungal drugs. Therefore, there is an urgent need for new antimycotic drugs with improved efficacy and reduced toxicity. The development of new antifungals based on antimicrobial peptides from various sources is considered a promising alternative. In this study, we examined the in vitro anti-yeast activity of the human cathelicidin peptides LL-37 against clinical strains of *C. auris* alone and in combination with different antifungal drugs by broth microdilution assay. To understand the antifungal mechanism of action, cell envelopes, cell cycle arrest, and effect on oxidative stress enzymes were studied using standard protocols. The minimum inhibitory and fungicidal concentrations of cathelicidin LL-37 ranged from 25–100 and 50–200 µg/mL, respectively. A combination interaction in a 1:1 ratio (cathelicidin LL-37: antifungal drug) resulted in 70% synergy with fluconazole and 100% synergy with amphotericin B and caspofungin. Assessment of the *C. auris* membrane by using propidium iodide assay after exposure to cathelicidin LL-37 linked membrane permeabilization with inhibition of *C. auris* cell growth and viability. These results were backed up by scanning electron microscopy studies demonstrating that exposure with cathelicidin LL-37 caused *C. auris* cells to undergo extensive surface changes. Spectrophotometric analysis revealed that cathelicidin LL-37 caused oxidative stress in *C. auris,* as is evident from the significant increase in the activity of primary antioxidant enzymes. In addition, cathelicidin LL-37 inhibited the cell cycle and accumulated cells in the S phase. Therefore, these results specify the potential of cathelicidin LL-37 for developing a new and effective anti-*Candida* agent.

## 1. Introduction

*Candida auris* is an emerging species of genus *Candida* that causes invasive infections in humans. Presently, *C. auris* has been reported as a multidrug-resistant nosocomial fungal pathogen that causes several outbreaks worldwide, and it is emerging as a menace to healthcare settings worldwide [1]. In the past decade, *C. auris* has been isolated from various clinical samples such as wounds, skin, body fluids, and mucocutaneous surfaces [2]. According to previous studies, severely ill patients in hospitals, especially in intensive care units (ICUs), are the primary victim of bloodstream infections caused by *C. auris*, with a high mortality rate of 30–60% [3,4]. Furthermore, *C. auris* has also been reported to have high antifungal drug resistance, with elevated MIC values to all three major antifungals (polyenes, azoles, and echinocandins) therefore narrowing the therapeutic options [5]. Distinct from species of *Candida*, *C. auris* has a unique ability to survive in a hostile environment outside the host, such as dry environmental surfaces, high temperature, and high salinity, which suggests that the pathogenic attributes of this *Candida* species facilitate adapting and persisting in unfavorable settings. Owing to its high tendency to cause outbreaks, misidentification, and resistance to antifungals, *C. auris* presents a global threat for immunocompromised patients in healthcare settings. The emergence of pan-resistant isolates of *C. auris* in some parts of the world has challenged the current therapeutic regimen, the increased mortality rates, and the adeptness of the pathogen to persist and survive in hospital settings has further complicated the scenario. Therefore, there is a pressing need to discover and develop alternative antifungal drugs to combat such deadly infections [6]. 

In past decades, various novel strategies for combating fungal infections have been explored. Organic compounds, including vitamin A and its derivative retinoids, have recently been reported to possess antifungal activity in preventing superficial and systemic fungal infections [7,8]. Antimicrobial peptides (AMPs) and synthetic derivatives encourage antimicrobial activity against multiple pathogens. AMPs, commonly named host defense peptides, play a vital role in innate immune response and have been widely reported in various microorganisms and humans [9]. Besides their antimicrobial activity, they are also identified as a robust immune modulator in the host [10]. Recently, AMPs have drawn the interest of researchers as a potential antimicrobial candidate as they display various important characteristics such as low cytotoxicity and adverse effect in a host, antimicrobial activity against various microorganisms, and less chance of triggering resistance mechanisms in microorganisms [11]. Besides antimicrobial peptides, a combination of drugs has already reached clinics to treat different resistant infections and has been declared a new weapon to fight multidrug resistance [12]. 

In mammals, AMPs belong to 2 families: cathelicidin and defensin; however, hCAP18, LL-37, and FALL39 are few cathelicidin reported in humans and are mainly located in the secondary granules of neutrophils [13,14]. Furthermore, the activated form of cathelicidin LL-37 is secreted from macrophages or monocytes and various epithelial cells [15,16]. Besides antimicrobial properties facilitated by membrane destabilization ability, cathelicidin LL-37 plays a critical role in mucosal defense against invasive pathogenic infections [17]. Additionally, they are also involved in processes such as, for instance, tissue regeneration, secretion of cytokine, angiogenesis, and preventing apoptosis in neutrophils [18]. However, the high cost involved in large-scale production, proneness to proteolysis, ability to trigger an autoimmune response and ability to stimulate growth in some cancer cells have restricted the use of cathelicidin LL-37 and other AMPs prospective drugs [19,20]. Nonetheless, natural peptides act as a scaffold for developing novel, potential, and affordable treatment options.

Therefore, in the quest to develop promising anticandidal therapeutics, the present study evaluated the antifungal potency of cathelicidin LL-37 individually and combined with 3 common antifungal drugs against clinical conditions isolates of *C. auris*. The effect of cathelicidin LL-37 on cell cycle progression and cell membrane integrity in *C. auris* was also evaluated.

## 2. Materials and Methods

### 2.1. Candida Strains and Growth Conditions

The present study utilized 10 clinical strains of *C. auris*, and the details are listed in Table 1. All of the clinical strains were preserved in the department at −80 °C as glycerol stocks. For the experimental procedure, *C. auris* strains were revived from glycerol stock and maintained on the YPD agar plates.

### 2.2. Antifungal Susceptibility Profiling

The MIC of cathelicidin LL-37 (Thermo Fisher Scientific, Eugene, OR, USA), amphotericin B, fluconazole, and caspofungin against *C. auris* strains (*n* = 10) was assessed by broth microdilution assay recommended in the standard M27 document (4th ed.) presented by CLSI with appropriate adjustments. Briefly, stock solutions of test peptide and respective antifungal drugs were prepared by using DMSO, and test concentrations ranged from 200–0.1 µg/mL for LL-37, 16–0.008 µg/mL for AmB, 1000–0.5 µg/mL for FLZ, and 16–0.008 µg/mL for CAS. The plates were incubated at 37 °C for 24 h and were observed for growth inhibition compared to untreated growth controls. The MIC was visually determined as the lowest concentration of antifungal drugs inhibiting fungal growth. 

Similarly, after MIC, the MFC was checked by subculturing 10 µL from the wells that showed no turbidity on SDA agar. The plates were incubated at 37 °C for 24. MFC was recorded as the lowest concentration without growth. 

### 2.3. Combination Studies

Utilizing the checkerboard microdilution method, LL-37 was evaluated for its antifungal activity combined with other antifungal drugs [21]. Briefly, in a 96-well microtiter plate, an equal volume of LL-37 (50 µL) and antifungals (AmB, FLZ, and CAS) were dispensed into predefined wells, accommodating 100 µL per well. The test concentration of LL-37 ranged from 200–0.1 µg/mL, whereas the concentration of AmB and CAS ranged from 16–0.008 µg/mL and the concentration of FLZ ranged from 1000–0.5 µg/mL. After serial dilution, 100 μL inoculum of *C. auris* strains (0.5 McFarland) was added to each well, and the plates were incubated at 37 °C for 24 h. The experiment included 1% DMSO (negative), growth, and sterility controls. The minimum inhibitory concentration (MIC) values were documented based on visual observations. The in vitro synergistic effect was estimated by calculating the fractional inhibitory concentration indexes (FICIs) according to the below-mentioned formula:

$$\mathrm{FICI}={\mathrm{FIC}\;}_{\mathrm{Drug}}+{\mathrm{FIC}}_{\;\mathrm{Peptide}}=\frac{{\mathrm{MIC}}_{\mathrm{drug}}\;\mathrm{in}\;\mathrm{combination}}{{\mathrm{MIC}}_{\mathrm{drug}}\;\mathrm{alone}\;}+\frac{{\mathrm{MIC}}_{\mathrm{peptide}}\;\mathrm{in}\;\mathrm{combination}}{{\mathrm{MIC}}_{\mathrm{peptide}}\;\mathrm{alone}\;}$$

The FICI values ≤ 0.5 reflects synergy, values > 4.0 reflects antagonism whereas, values between 0.5 and 1.0 reflects additive, and between 1.0 and 4.0 is indifferent [21,22].

### 2.4. Cell Viability and Cell Count Assay

The evaluation of the fungicidal potential of LL-37 against *C. auris* MRL6057 (multidrug-resistant strain) was conducted using Muse^TM^ Count and Viability assay kit. The procedure recommended by the manufacturer was adopted to perform the assay. Briefly, yeast cells were exposed to different concentrations of the LL-37 (MIC and MFC) for 4 h at 37 °C. Followed by centrifugation and washing with fresh PBS, and after that, 20 µL of aliquot was added to 380 µL of Count & Viability reagent, followed by incubation for 5 min at RT. Muse^TM^ cell analyzer was used to analyze the viability and cell count of yeast cells exposed and unexposed to various conditions. Alternatively, cells exposed to H_2_O_2_ (10 mM) were used as positive controls, while untreated healthy cells were taken as negative controls.

### 2.5. Time-Kill Kinetics

Time-kill kinetics of LL-37 against the multidrug-resistant strain of *C. auris* was performed, and the method was adopted from Klepser and co-workers [22,23]. Briefly, yeast cells were adjusted to a density of 1 × 10^6^ CFU/mL and treated with different test peptide concentrations (MIC and MFC), followed by incubation at 37 °C, 200 rpm for 48 h. At predetermined time intervals (0, 2, 4, 6, 8, 12, 24 and 48 h), aliquots of 100 µL were dispensed, washed with sterile PBS, and serial dilutions were prepared, from which 20 µL were plated onto Sabouraud dextrose agar (SDA; Merck, RSA) plates and incubated for 24 h at 37 °C. Post-incubation colonies were counted, colony-forming unit (CFU) was determined, and the result was recorded as log10 CFU/mL. A growth control, with healthy *C. auris* cells and no peptides, was also included in the study.

### 2.6. Effect of Cathelicidin LL-37 on Antioxidant Enzymes

The effect of LL-37 on vital antioxidant enzymes of *C. auris* was investigated in the present study. A single colony of *C. auris* MRL6057 cells was inoculated in Sabouraud dextrose broth (SDB; Merck, RSA) and incubated at 37 °C, 200 rpm for 6–8 h (mid-log phase); after that, the cells were washed with PBS and later on exposed to different concentrations of test peptide (MIC and MFC) for 4 h. Cell-free extract (CFE) was prepared as described elsewhere [23] and was used for the estimation of antioxidant enzymes and lipid peroxidation (LPO) in *C. auris* MRL6057.

### 2.7. Antioxidant Assays 

The estimation of catalase (CAT), superoxide dismutase (SOD), and glutathione peroxidase (GPx) was carried out by following the method previously described [24]. The glutathione reductase (GLR) and glutathione transferase (GST) estimation was carried out as described elsewhere [25,26,27]. Yousuf et al. method was adopted to estimate lipid peroxidation (LPO) [24]. 

### 2.8. Effect of Cathelicidin LL-37 on C. auris Cell Cycle

The impact of LL-37 on the *C. auris* cell cycle was evaluated using MuseTM Cell Analyzer. The instructions provided by the manufacturer were adopted for the current analysis. Briefly, a single colony of *C. auris* MRL6057 cells was inoculated in SDB and incubated at 37 °C, 200 rpm for 6–8 h, washed with PBS, and then exposed to different concentrations of test peptide (MIC and MFC) for 4 h. Post-incubation cells were again washed with PBS, and the pellet was fixed with ice-cold 70% ethanol (1 mL; Sigma Aldrich Co., St. Louis, MO, USA). After that, Muse™ Cell Cycle reagent was mixed with the fixed cells in equal volume incubated for half an hour in the dark at room temperature. Additionally, cells exposed to H_2_O_2_ (10 mM) were used as positive controls, while untreated healthy cells were taken as negative controls for the experiment.

### 2.9. Effect of Cathelicidin LL-37 on C. auris Membrane Integrity 

The effect of LL-37 on plasma membrane integrity of *C. auris* was examined by Propidium Iodide (Sigma-Aldrich) staining method as it is used as a universal marker for studying plasma membrane permeability [28]. The experiment was conducted as previously described [29], with modifications. Briefly, *C. auris* MRL6057 cells were inoculated in SDB and incubated at 37 °C, 200 rpm for 24 h. Post-incubation cells were washed twice with sterile PBS, resuspended in SDB (turbidity was adjusted to 0.5 McFarland), and exposed to an appropriate concentration of LL-37 (MIC and MFC) for 4 h. Both positive (exposed to H_2_O_2_, 10 mM) and negative controls were included in the study. The cells were then washed twice with PBS and stained with PI (30 µM), and incubated for 30 min at room temperature in the dark. After incubation, cells were rewashed with PBS, and the pellet was resuspended in PBS (250 µM), and 10 µM of the sample was used for fluorescence microscopy (Zeiss Laser Scanning Confocal Microscope (LSM) 780 and Airyscan (Carl Zeiss, Inc. Jena, Germany).

### 2.10. Scanning Electron Microscopy 

Scanning electron microscopy (SEM) was used to study the effect of LL-37 on the cell morphology of *C. auris* MRL6057. The yeast cells were adjusted to a density of 1 × 10^6^ CFU/mL and treated with different concentrations (MIC and MFC) of the test peptide. Followed by incubation at 37 °C, 200 rpm for 48 h. h. Post incubation, aliquots of 100 µL were withdrawn, washed with PBS, and fixed with 2.5% glutaraldehyde for 2 h at room temperature. Afterward, fixed cells were rewashed with PBS and were subjected to gradient dehydration with ethanol (4%, 10 min; 60%, 10 min; 80%, 10 min, and 100%, 20 min). Later, 20 µM aliquots of fixed and dehydrated cells were used to prepare slides and subjected to critical point drying, carbon-coated, and observed under the SEM (Zeiss Gemini 2 Crossbeam 540 FEG SEM). 

### 2.11. Statistics

Graph Pad Prism version 9.1.0 was used for statistical analysis. All of the experiments were conducted in triplicate, and data were presented as the average of 3 independent experiments (mean ± SD), and statistical significance was determined using the Student-*t*-test (*p*-value <  0.05).

## 3. Results

### 3.1. Antifungal Potential of Cathelicidin LL-37 against C. auris Isolates

Human cathelicidin LL-37 exhibited potent antifungal activity against all ten clinical strains of *C. auris*. The MIC values ranged from 25 to 100 µg/mL, whereas the MFC values were 3-fold higher than their corresponding MIC values (Table 2). Therefore, suggesting that LL-37 has fungicidal activity against clinical strains of *C. auris*. Whereas the MIC values for AmB ranged from 0.125 to 4 µg/mL, for FLZ, it ranged from 16 to 500 µg/mL, and in the case of CAS, the values ranged from 0.25 to 2 µg/mL (Table 2). According to the published tentative MIC Breakpoints for *C. auris* [26,30], 8strains were found resistant to FLZ (MIC ≥ 32 µg/mL), 5 strains were resistant to AmB (MIC ≥ 2 µg/mL), and only 1 strain was resistant to CAS (MIC ≥ 2 µg/mL). Based on this result, *C. auris* MRL6057 was the most resistant, with the highest MIC values against all of the antifungal drugs; therefore, it was selected to interrogate the fungicidal effect of the LL-37 further. 

### 3.2. Antifungal Activity of Cathelicidin LL-37 in Combination with Standard Antifungal Drugs 

Based on the FIC indices, it can be postulated that LL-37 in combination with FLZ, AmB, and CAS presented has a synergic effect against all of the tested clinical strains of *C. auris*. The FICI values ≤0.5 for variety suggested that LL-37 and other standard antifungal drugs synergistically inhibited the growth of *C. auris* (Table 3). When LL-37 was combined with FLZ synergistic effect was observed in 80% of *C. auris* strains whereas, in combination with AmB and CAS, a synergistic effect was observed in all the clinical strains of *C. auris*. Furthermore, no antagonistic activity was detected for any of the tested combinations. The combination lowered the MIC values of both antifungal drugs and peptides by around 4 to 8-fold compared to their respective MIC values. Therefore, it can be concluded that LL-37 acts synergistically and tends to lower the dosage of commonly used antifungals used to treat Candida infection and thereby reduce the toxicity in the host. 

### 3.3. Cathelicidin LL-37 Impedes the Growth and Viability of C. auris 

**Figure 1:**
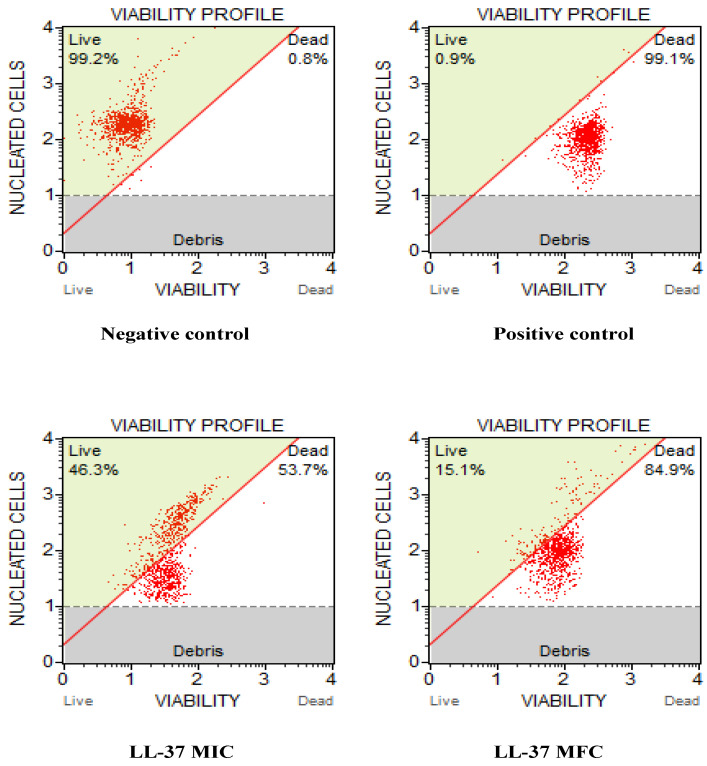
Effect of cathelicidin LL-37 on cell count and viability of *C. auris* MRL6057 cells. The figures represent the untreated yeast cells as a negative control, cells exposed to 10 mM H_2_O_2_ as a positive control, and cells exposed to different concentrations (MIC and MFC) of LL-37.

The results obtained for cell count and viability were represented in the graph from Muse^TM^ Cell Analyzer (Figure 1). The results showed that exposure to LL-37 reduced the growth and survival of *C. auris* cells. The untreated control was mainly composed of live cells (99.2%), distinct from the positive control (0.9%). With the increasing concentration of LL-37, there was a drastic decrease in the percentage of live *C. auris* cells; at MIC value, the percentage of live cells was 46.3% which further reduced to 15.1% at MFC. Therefore, these results further reveal that LL-37 inhibits the growth and survival of *C. auris* cells and therefore should be further investigated for its mode of antifungal action.

### 3.4. Time-Kill Kinetics of Cathelicidin LL-37 in C. auris Cells 

**Figure 2:**
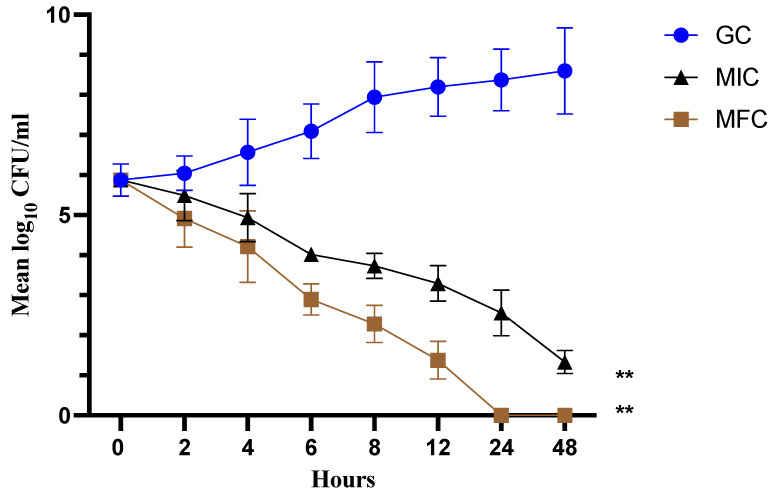
Time-kill kinetics. The graph represents a fungicidal property of cathelicidin LL-37 at MIC and MFC concentration against *C. auris* MRL6057. (** *p* < 0.01 compared with GC).

The time-dependent kill curve for cathelicidin LL-37 at different concentrations (MIC and MFC) against *C. auris* MRL6057 was prepared. The fungicidal effect of the test peptide was defined as ≥3 log10 decrease in CFU/mL (≥99.9% killing) from the initial inoculum. The cathelicidin LL-37 displayed complete killing of *C. auris* cells within 8 h at MFC and 24 h at MIC value (Figure 2). This correlated with the antifungal susceptibility results and demonstrated the dose-dependent fungicidal activity of cathelicidin LL-37 against *C. auris*.

### 3.5. Cathelicidin LL-37 Modulates the Activity of Antioxidant Enzymes in C. auris 

**Figure 3:**
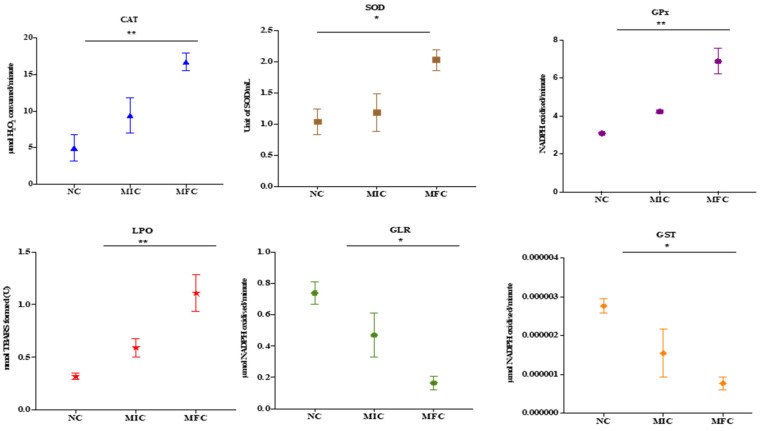
Antioxidant enzyme activity of *C. auris.* Effect of cathelicidin LL-37 (MIC and MFC) on the activity of various antioxidant enzymes present in *C. auris*. Untreated cells are considered as negative control (NC). Asterisks indicate significant difference. (* *p* < 0.05 compared with NC; ** *p* < 0.01 compared with NC).

The effect of cathelicidin LL-37 over the antioxidant enzymes of *C. auris* MRL6057 is shown in Figure 3. With increasing LL-37 concentrations, catalase activity increased. For untreated negative control, the average values for CAT activity were 5.01 μmol of H_2_O_2_ consumed/min; however, after exposure to LL-37 the CAT activity increases to 9.41 and 16.71 μmol of H_2_O_2_ consumed per min against MIC and MFC, respectively. 

Similarly, exposure to LL-37 also affected SOD activity in the *C. auris*, and both MIC and MFC measurements indicated an increase in enzyme activity. In contrast, the measurement for the negative control resulted in a value of 1.03 units/mL. Conversely, GPx activity also increased. Enzymatic activity of 3.08 mol NADPH was observed in the negative control. Nevertheless, for MIC and MFC, the calculated NADPH oxidization rates were 4.23 and 6.89 mol/min, respectively.

The enzyme activity of GST and GLR, on the other hand, decreased. In addition, GST activity in cells exposed at MIC and MFC was 1.54 × 10^−6^ & 0.77 × 10^−6^, respectively, and negative control 2.76 × 10^−6^ μmol of CDNB conjugate formed/min. Compared to this, the GLR activity was 0.47 and 0.16 μmol of NADPH oxidized/min at their respective MIC and MFC concentrations, compared to the negative control (0.74 μmol of NADPH oxidized/min) under similar conditions. 

Estimation of lipid peroxidation in *C. auris* MRL6057 was based on the generation of TBARS. The results showed that TBARS formation rates gradually increased after exposure to citral. The increase in TBARS formed in exposed *C. auris* cells was 00.59 and 1.1 nmol against MIC and MFC, respectively, whereas the value for the negative control was 0.31. It was shown that the test compound modulated the critical parameters of oxidative stress in *C. auris*. As a result, the levels of LPO and the activities of defense enzymes deviated from the normal trend.

### 3.6. Cathelicidin LL-37 Arrest Cell Cycle in S Phase in C. auris 

**Figure 4:**
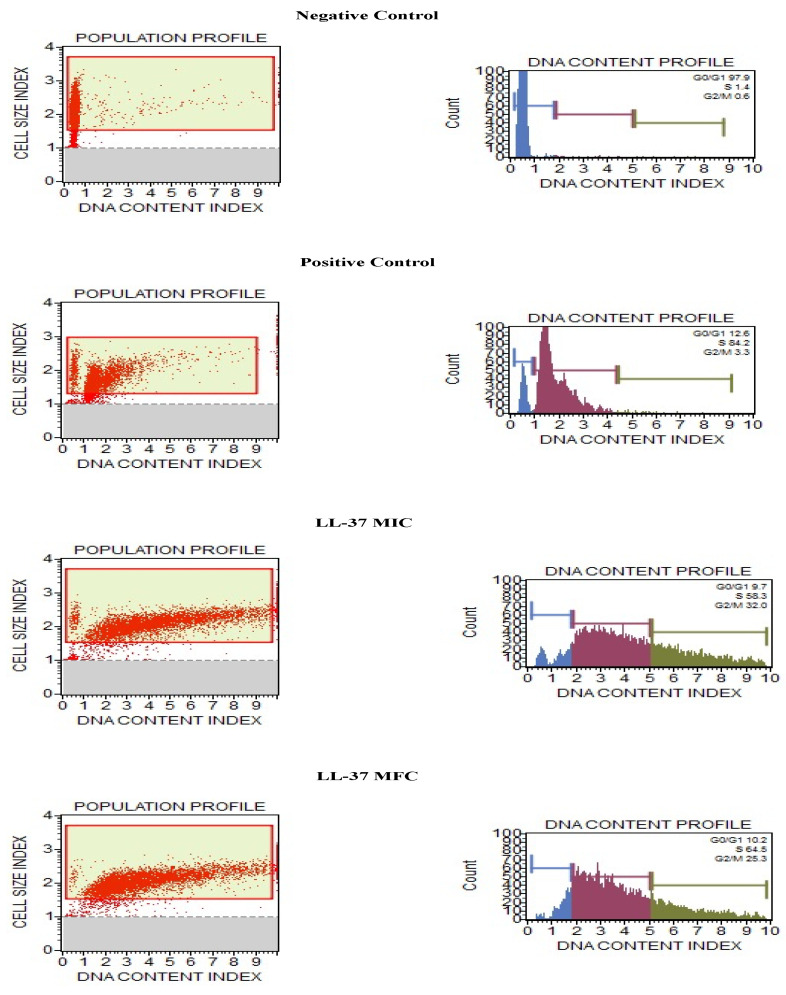
Cell cycle study in *C. auris* by Muse™ Cell Analyzer. Effect of different concentrations of cathelicidin LL-37 (MIC and MFC) on cell cycle progression in *C. auris* MRL6057. Cells exposed to H_2_O_2_ (10 mM) were considered positive control, whereas untreated cells are negative control.

Our investigation aimed to discover antifungal compounds that act specifically against fungi and further enhance our findings. Thus, we measured the effect of the cathelicidin LL-37 peptide over the *C. auris* MRL6057 cell cycle. Therefore, distorted and deviated cell cycle trends in exposed cells would indicate that cathelicidin LL-37 is disrupting the cell cycle and possibly arresting the cell cycle at different stages. To confirm cell cycle arrest in *C. auris*, fluorescence intensity from propidium iodide DNA was used to quantitatively estimate the DNA content present in various stages of cell cycle cells. As a result, the negative control had cells in the G0/G1 phase 97.9% of the time, 1.4% in the S phase, and 0.6% in the G2/M phase, respectively. Among the cells in the positive control, 12.6%, 84.2%, and 3.3% were found in G0/G1, S, and G2/M phases, respectively. At MIC values, the distribution of cells in different stages was 9.7% in G0/G1, 58.3% in S, and 32% in G2/M. Similarly, at MFC value, 10.2% of cells were found in G0/G1, 64.5% in the S phase, and 25.3% in the G2/M phase. These results reinforce cathelicidin LL-37 exposure had a pronounced effect on the cell cycle and compelled the cells to get arrested in the S phase (Figure 4).

### 3.7. Effect of Cathelicidin LL-37 on C. auris Membrance Integrity 

**Figure 5:**
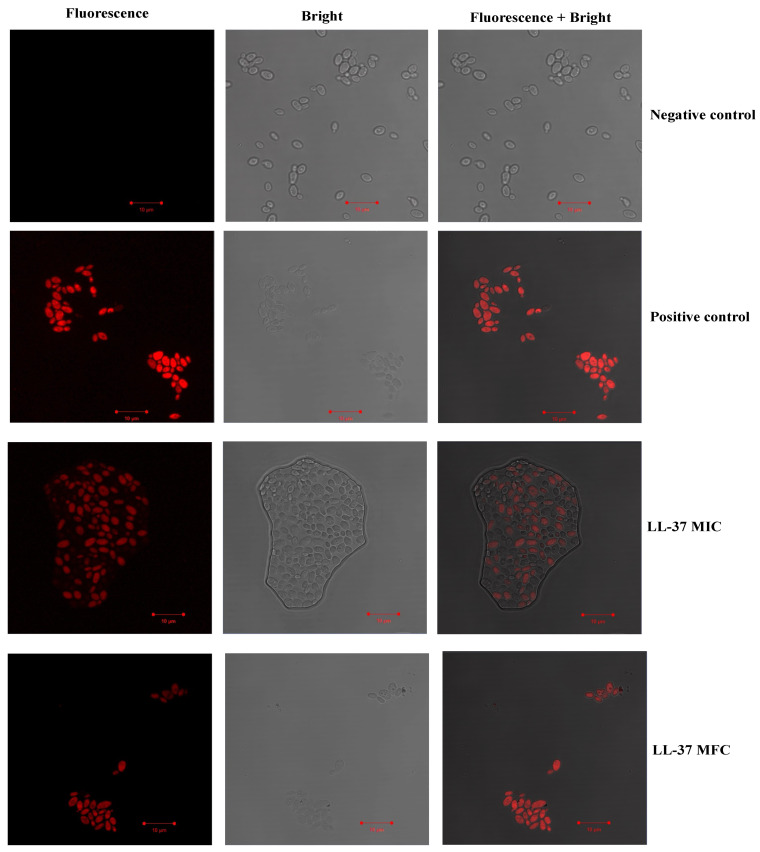
The uptake of PI by *C. auris* MRL 6057. A range of concentrations of cathelicidin LL-37 treated to yeast cells (MIC and MFC). Untreated cells were used to examine *C. auris* plasma membrane integrity, while cells treated with H_2_O_2_ showed compromised membrane integrity leading to PI uptake into cells.

The effect of test peptide on membrane integrity of *C. auris* MRL6057 was investigated by using PI. Exposure to cathelicidin LL-37 resulted in disruption of the plasma membrane in *C. auris* cells; as a result, PI diffusion was observed into the cells, and therefore, an increasing number of PI-positive yeast cells was observed under microscopic study (Figure 5). A higher uptake of PI was observed with the increasing concentration of cathelicidin LL-37; maximum uptake was observed at MFC values, followed by lower concentrations. 

### 3.8. Effect of Cathelicidin LL-37 Cell Morphology of C. auris MRL6057

**Figure 6:**
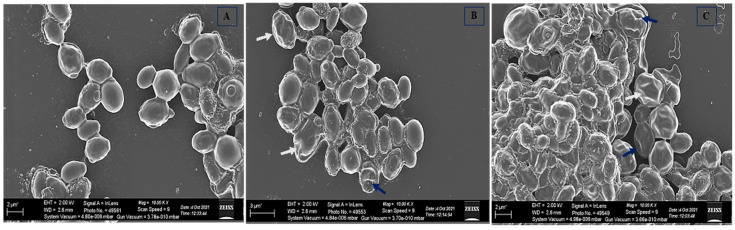
SEM images of *C. auris* MRL6057. (**A**) Untreated control; Yeast cells exposed to cathelicidin LL-37 at different contractions, (**B**) MIC and (**C**) MFC. Cell surface wrinkles, breakage, and deformities are represented by white arrows, while blue arrows indicate leakage of intracellular content.

The effect of cathelicidin LL-37 on the cellular architecture of *C. auris* was monitored by SEM, and the results are displayed in Figure 6. The untreated yeast cells possessed a uniform three-dimensional morphology and were healthy with a smooth and unbroken surface (Figure 6A). Yeast cells incubated with cathelicidin LL-37 were characterized by various sizes, irregular shapes, squeezed, depressed surfaces, and the release of intracellular components. These features indicate that the cells are in the process of degrading (Figure 6B,C). Cathelicidin LL-37 produced profound depressive effects in yeast cells due to its cytocidal effects. Therefore, our results from PI uptake and SEM assay reflect that cathelicidin LL-37 also disrupts the integrity of the fungal cell membrane resulting in cell death.

## 4. Discussion

Antimicrobial peptides are key elements of innate immunity, and more than hundreds of such peptides have been discovered in different human tissues. Among them, cathelicidin LL-37 is the most explored one, and there has been an increasing interest in designing new derivatives of cathelicidin LL-37. Cathelicidin LL-37 has potential clinical applications; however, not limited to antibacterial, antibiofilm, antiviral, antifungal, immune-modulating, and anticancer peptides. Different derivatives of LL-37 have been engineered into 17BIPHE2, an antimicrobial, antibiofilm, and anticancer peptide that is stable, selective, and potent. Furthermore, 17BIPHE2 and SAAP-148 can inhibit ESKAPE pathogen-induced biofilms and be used in vivo as topical antibiofilm [31]. In a randomized controlled trial conducted by Gronberg and co-workers (2014), the safety and dose-response efficacy of the human synthetic peptide LL-37 for treating hard-to-heal Venous leg ulcers was studied. The results suggested that topical treatment with LL-37 for chronic leg ulcers was safe and well-tolerated [32].

Owing to the increasing resistance among clinically relevant pathogenic species of Candida, there is an urgent need to develop efficient and safe antifungal drugs. Decreasing susceptibility to antifungals, namely, azoles and echinocandins, has complicated the treatment strategies, and the management of fungal infections has become challenging [33]: antimicrobial peptides, both naturally existing and synthetic, offer an encouraging platform for advancing new candidacidal drugs. The antibacterial activity of human cathelicidin LL-37 against various multidrug-resistant bacterial pathogens has already been demonstrated [34,35,36]. However, the antifungal activity of naturally existing and synthetic antimicrobial peptides has been explored only by a few researchers [37,38]. The LL-37 has been found active against planktonic cells and prevented biofilm formation in Candida species (MIC varied from 4–≥ 64 μM) [39]. The present work demonstrated the anti-Candida activity of human cathelicidin peptides (LL-37) against various clinical strains of *C. auris*, and the antifungal potential was compared with standard antifungal drugs. Cathelicidin LL-37 was effective against both sensitive and resistant strains of *C. auris*. 

In clinical practice, using a combinatorial approach for treating invasive candidiasis is very common [40]. Similarly, a combination of different classes of antifungals (echinocandins plus azoles or AmB) has been widely recommended for treating bloodstream infections caused by *C. auris* [41,42]. Therefore, combining antimicrobial peptides with standard antifungal drugs can be a boon to antifungal regimens [43]. Researchers have reported in vitro synergistic effect of various AMPs with antimycotic drugs against clinical strains of C. albicans [44]. In vivo results against bloodstream Candida infections were found to be promising [45]. Our results also supported this hypothesis, and a high level of synergism was observed in almost all the antifungal-LL-37 combinations.

The cathelicidin LL-37 has been found to possess antifungal activity against various Candida species by disrupting the yeast cell membrane through efflux of ATP and proteins and interacting with cell wall components [39]. Researchers have reported antifungal activity of a group of LL-37 based peptides against various fungal species at low concentrations of <1 μM. In this study, the quantitative estimation performed by using Muse Count & Viability kit confirmed the fungicidal activity of LL-37 against *C. auris* MRL6057, which is a multidrug-resistant strain. The Muse reagents is a mixture of two DNA binding dyes that enables differential staining of viable and non-viable cells based on their permeability to the dyes.

Due to the increase in multidrug resistance among fungal pathogens against commonly used antimycotic drugs, the treatment strategy is becoming a puzzle worldwide, leading to an immense effort to develop new and more effective antifungal agents. Among the available options, the most promising ones are AMPs [46]. Their presence is reported in all biological organisms, functioning by physically infiltering and promptly disrupting the microbial cell membrane [47]. Therefore, the likelihood of developing microbial resistance toward AMPs is significantly less. Owing to these properties, AMPs are explored as a potential candidate for antifungal drug development. The current results agree with the anti-Candida potential of cathelicidin LL-37, showing is fungicidal effect against drug-resistant *C. auris* strain.

Antioxidant enzymes play a critical role in regulating immune coping mechanisms, antimicrobial resistance, and other virulence attributes [48]. For instance, exposure to foreign substances, therapeutic agents are known for disrupting microbial cell membranes tend to elevate reactive oxidant species (ROS) levels and trigger oxidative stress within the cell. Resulting inactivation of a series of events such as DNA breakdown, lipid oxidation, and various physical and molecular changes, leading to cell death. Healthy cells are equipped with unique systems that help balance ROS generation with different antioxidant defense schemes comprised of enzymatic and non-enzymatic scavengers such as antioxidant enzymes. Based on their mechanism of action, antioxidant enzymes are classified into primary, secondary, and tertiary antioxidants. Catalase (CAT), superoxide dismutase (SOD), and glutathione peroxidase (GPx) are primary antioxidants as they inactivate ROS into its intermediates (O_2_−–SOD→ H_2_O_2_–CAT → H_2_O + O_2_). The primary antioxidant enzymes are continuously supplied with glutathione and nicotinamide adenine dinucleotide phosphate (NADPH) by secondary antioxidant enzymes such as glutathione reductase (GR) and glutathione-S-transferase (GST). [49]. As ROS levels increase in aerobic organisms, SOD and CAT become active, which are essential for the defense against oxidative stress by consuming H_2_O_2_ and maintaining H_2_O_2_ [50,51]. However, if, for some reason, the antioxidant detoxification phenomenon fails and the level of ROS goes beyond the threshold level, then this leads to the onset of oxidative stress. Therefore, in this study, the effect of cathelicidin LL-37 on antioxidant enzymes of *C. auris* was evaluated. Our results presented that cathelicidin LL-37could modulate the activity of crucial antioxidant enzymes, thereby inducing ROS production in a concentration and time-dependent manner, which leads to cell death. Therefore, it could be inferred that the generation of oxidative stress might be involved in the action mechanism of cathelicidin LL-37.

Increased ROS within an organism can cause oxidative stress and is caused by external factors such as antifungal drugs, which cause the membrane to become permeable. As a result of oxidative stress, DNA is disorganized, lipids are oxidized, and cellular structures are changed [52]. In response to ROS, LPO is one of the inevitable symptoms of oxidative stress. The production of lipid peroxides, which ROS commonly induces, can cause bilayers to deform and membranes to lose functionality. TBA reactive substances (TBARS) buildup in the cytoplasm represents this lipid damage [49]. In the TBARS assay, the reaction of MDA with TBA is spectrophotometrically measured under acidic conditions and heat [53]. This method provided evidence of an increase in LPO in this study. Compared with the untreated *C. auris* cells, the quantity of TBARS formed increased after being treated with cathelicidin LL-37. Therefore, this study proves that exposure to cathelicidin LL-37 causes LPO in *C. auris* strain, indicative of membrane disintegration. These observations are consistent with the results of previous studies that examined the LPO in the context of other Candida species [49].

Cathelicidin LL-37 inhibits the cell cycle in *C. auris* MRL6057, further strengthening its anti-Candida activity. The cell cycle is a necessary process for cell proliferation; therefore, if a distorted percentage of cells are present during various phases of the cell cycle compared with healthy, growing cells, this leads to the arrest of the cell cycle. Consequently, DNA content change was quantified during different cell cycle phases by evaluating the fluorescence intensity produced by DNA labeled with PI, directly proportional to a particular cell cycle phase. Cell cycle arrest is a vial mechanism adopted for the survival of eukaryotic cells. It confirms cellular integrity during growth and replication, curtailing the chances of unusual mutations and unfavorable cell growth [54]. Hence, it could be concluded that the exposure of cathelicidin LL-37 inhibits DNA synthesis in yeast cells. The *C. auris* cells accumulated in the S phase of the cell cycle since damaged DNA could not prevent mutations.

The microbial plasma membrane is vital for the growth and survival of cells because it acts as an obstruction to external environmental stresses. Therefore, compounds aiming at the fungal plasma membrane could be considered a possible lead for developing new antifungal drugs with increased efficacy. PI can enter disrupted cell membranes and is used as a marker nucleic acid. The process, namely necrosis, causes damage to the plasma membrane and allows PI to enter the cells, resulting in red fluorescence. The fluorescence due to PI in the cells indicates a defect in the cell plasma membrane [54]. Previously, researchers have reported the mechanism of action of AMPs, and their antimicrobial property is attributed to its membrane permeabilization tendency [55,56]. Therefore, we speculate that the plasma membrane disruption after exposure to cathelicidin LL-37 is the mode of action accompanied by the generation of oxidative stress in *C. auris* MRL6057.

## 5. Conclusions

In conclusion, our results reinforce that cathelicidin LL-37 has potent antifungal activity alone and in combination with traditional antifungal drugs. Insight mechanisms revealed that cathelicidin LL-37 disrupts cell membrane integrity, triggers oxidative stress, and arrests the cell cycle in S-phase. In vivo studies demonstrating the additional mechanisms and cytotoxicity on mammalian cells of this antimicrobial peptide will be required to establish these claims further. Altogether, these results support the notion that cathelicidin LL-37 is a potential candidate in developing a new antifungal drug to combat serious fungal infections.

## Figures and Tables

**Table 1:** Clinical strains of *C. auris* used in the study.

Study ID	Clinical ID
CAU-01	MRL 3499
CAU-02	MRL3785
CAU-03	MRL4000
CAU-04	MRL2921
CAU-05	MRL5762
CAU-06	MRL5765
CAU-07	MRL6277
CAU-08	MRL6065
CAU-09	MRL6057
CAU-10	MRL6173

**Table 2:** Antifungal susceptibility profile of cathelicidin LL-37 and other antifungal drugs against clinical strains of *C. auris*.

*C. auris*	MIC/MFC (µg/mL)							
	Cathelicidin LL-37		Amphotericin B		Caspofungin		Fluconazole	
	MIC	MFC	MIC	MFC	MIC	MFC	MIC	MFC
CAU-01	50	100	0.5 (S)	1.0	0.25 (S)	0.5	16.0 (S)	FS
CAU-02	25	50	0.12 (S)	0.5	0.25 (S)	0.5	16.0 (S)	FS
CAU-03	100	200	2.0 ®	4.0	0.25 (S)	0.5	250.0 (R)	FS
CAU-04	50	100	2.0 (R)	4.0	0.5 (S)	1.0	250.0 (R)	FS
CAU-05	100	200	2.0 (R)	4.0	0.25 (S)	0.5	500.0 (R)	FS
CAU-06	50	100	2.0 (R)	4.0	0.25 (S)	0.5	500.0 (R)	FS
CAU-07	25	50	0.5 (S)	1.0	0.25 (S)	1.0	125.0 (R)	FS
CAU-08	100	200	1.0 (S)	2.0	0.25 (S)	0.5	125.0 (R)	FS
CAU-09	50	100	4.0 (R)	8.0	2.0 (R)	4.0	125.0 (R)	FS
CAU-10	50	100	0.25 (S)	0.5	0.25 (S)	0.5	(R)	FS

S, sensitive; R, resistance. Classification based on CDC guidelines; FLZ (S < 32 µg/mL; R ≥ 32 µg/mL); AmB (S < 2 µg/mL; R ≥ 2 µg/mL); CAS (S < 2 µg/mL; R ≥ 2 µg/mL). FS: fungistatic.

**Table 3:** In vitro antifungal activity of cathelicidin LL-37 in combination with standard antifungal drugs against clinical strains of *C. auris*.

Test Agent	Strains	MIC Alone (µg/mL)		MIC in Combination (µg/mL)		FICI	INT
		MIC-A	LL-37-A	MIC-B	LL-37-B		
LL-37-FLZ	CAU-01	16	50	16	3.125	1.06	IND
	CAU-02	16	25	16	3.125	1.13	IND
	CAU-03	250	100	63	12.5	0.38	SYN
	CAU-04	250	50	63	12.5	0.50	SYN
	CAU-05	500	100	63	12.5	0.25	SYN
	CAU-06	500	50	63	12.5	0.38	SYN
	CAU-07	125	25	63	12.5	0.63	ADD
	CAU-08	125	100	32	6.25	0.32	SYN
	CAU-09	125	50	32	6.25	0.38	SYN
	CAU-10	32	50	8	1.56	0.27	SYN
LL-37-AmB	CAU-01	0.5	50	0.125	0.78	0.27	SYN
	CAU-02	0.12	25	0.031	0.195	0.26	SYN
	CAU-03	2	100	0.25	1.56	0.14	SYN
	CAU-04	2	50	0.5	3.125	0.31	SYN
	CAU-05	2	100	0.25	1.56	0.14	SYN
	CAU-06	2	50	0.5	3.125	0.31	SYN
	CAU-07	0.5	25	0.062	0.39	0.14	SYN
	CAU-08	1	100	0.25	1.56	0.27	SYN
	CAU-09	4	50	0.5	3.16	0.20	SYN
	CAU-10	0.25	50	0.031	0.20	0.13	SYN
LL-37-CAS	CAU-01	0.25	50	0.062	0.39	0.26	SYN
	CAU-02	0.25	25	0.062	0.39	0.26	SYN
	CAU-03	0.25	100	0.031	0.195	0.13	SYN
	CAU-04	0.5	50	0.062	0.39	0.13	SYN
	CAU-05	0.25	100	0.031	0.195	0.13	SYN
	CAU-06	0.25	50	0.031	0.195	0.13	SYN
	CAU-07	0.25	25	0.031	0.195	0.13	SYN
	CAU-08	0.25	100	0.062	0.39	0.25	SYN
	CAU-09	2	50	0.5	3.125	0.13	SYN
	CAU-10	0.25	50	0.031	0.195	0.13	SYN

MIC is the median MIC of three independent experiments. MIC-A and MIC-B are the median MIC of the drug alone and in combination, respectively. LL-37-A and LL-37-B are the median MIC of the peptide alone and in combination, respectively. FICI, fractional inhibitory concentration index; presented as mean ±standard deviation. INT, interpretation; SYN, synergy; IN, indifferent; ADD, Additive.

## Data Availability

The data available is provided in this manuscript.
